# Effects of dipotassium glycyrrhizinate on wound healing

**DOI:** 10.1590/ACB360801

**Published:** 2021-10-08

**Authors:** Camila dos Santos Leite, Oscar César Pires, Danielle Gatti Tenis, Jussara Vaz Nascimento Ziegler, Denise Gonçalves Priolli, Thalita Rocha

**Affiliations:** 1 MSc, Fellow PhD degree. Multidisciplinary Research Laboratory, Universidade São Francisco (USF) - Bragança Paulista (SP), Brazil.; 2 PhD, Assistant Professor. Physiology and Pharmacology Laboratory – Universidade de Taubaté (UNITAU) – Taubate (SP), Brazil.; 3 Graduate student. Universidade São Francisco (USF) - Bragança Paulista (SP), Brazil.; 4 MSc. Multidisciplinary Research Laboratory - Universidade São Francisco (USF) - Bragança Paulista (SP), Brazil.; 5 PhD, Assistant Professor. Multidisciplinary Research Laboratory - Universidade São Francisco (USF) - Bragança Paulista (SP), Brazil.; 6 PhD, Assistant Professor. Multidisciplinary Research Laboratory - Universidade São Francisco (USF) - Bragança Paulista (SP), Brazil.

**Keywords:** Glycyrrhizic Acid, Wound Healing, Collagen, Models, Animal, Rats

## Abstract

**Purpose:**

Dipotassium glycyrrhizinate (DPG) has anti-inflammatory properties, besides promoting the regeneration of skeletal muscle. However, it has not been reported on skin wound healing/regeneration. This research aimed to characterize the effects of DPG in the treatment of excisional wounds by second intention.

**Methods:**

Male adults (n=10) and elderly (n=10) Wistar rats were used. Two circular wounds were excised on the dorsal skin. The excised normal skins were considered adult (GAN) and elderly (GIN) naïve. For seven days, 2% DPG was applied on the proximal excision: treated adult (GADPG) and elderly (GIDPG), whereas distal excisions were untreated adult (GANT) and elderly (GINT). Wound healing areas were daily measured and removed for morphological analyses after the 14^th^ and the 21^st^ postoperative day. Slides were stained with hematoxylin-eosin, Masson’s trichrome, and picrosirius red.

**Results:**

Histological analysis revealed intact (GAN/GIN) and regenerated(GANT/GINT/GADPG/GIDPG) skins. No differences of wounds’ size were found among treated groups. Epidermis was thicker after 14 days and thinner after 21 days of DPG administration. Higher collagen I density was found in GIDPG (14^th^ day) and GADPG (21^st^ day).

**Conclusions:**

DPG induced woundhealing/skin regeneration, with collagen I, being more effective in the first 14 days after injury.

## Introduction

Wound healing is characterized by cellular and molecular complex events, consisting of three phases–inflammatory, proliferative and remodelling–, and involves cell division, reepithelialization, neovascularization, collagen synthesis, remodelling and structural contraction^1^^,^ 2.

The healing mechanism occurs physiologically, but there are several local and/or systemic factors that can interfere negatively, for example: contamination, smoking, inadequate treatment, oxygen supply and deficient nutrients due to decreased perilesional vascularity, immunosuppressive disease, medications (corticosteroids, chemotherapy, radiotherapy) and nutritional deficiencies (vitamins B, C, D and E), and aging, regarding the individual’s clinical conditions and chronic diseases, such as cardiovascular diseases, diabetes mellitus and associated neuropathies^3^^-^ 7.

Biological aging is a process in which important physiological, morphological, and biochemical changes gradually occur. Loss of the arrangement between the elastic and collagen fibers in the dermal papillae or decrease in size and proliferation of keratinocytes in the basal stratum lead to epidermis thickness structural and functional changes, clearly identifiable in the elderly skin^5^. Specifically, the dermis can undergo progressive changes, with a thin thickness due to the fibroblasts rarefaction, collagen synthesis decreasing and elastic fibers fragmentation. Also, changes in the amount of water and glycosaminoglycans occur, creating spaces between elastic and collagen fibers. In addition, cell migration capability and vascularity reduce, and size and secretion of sebaceous and sweat glands decrease^5^. Those structural changes result in a thin and fragile skin in elderly, vulnerable to mechanical forces and contamination, as well as in a slow process of skin healing^5^^,^ 7.

In recent years, research has been carried out seeking for alternative methods or drugs to help, correct, improve, or accelerate the skin healing process. Thus, new compounds have been studied, aiming mainly to promote regeneration of the epidermis^8^ and dermis^8^^-^ 10.

*Glycyrrhiza glabra* L., known as licorice, has shown nutritional and pharmacological properties especially assigned to glycyrrhizin^11^. Another isolated compound, the glycyrrhizic acid (GA), has been used regarding its antitumor, antiallergic, antibiotic and anti-inflammatory properties^12^^-^ 14. Dipotassium glycyrrhizinate (DPG), a secondary product of GA, has antiallergic, antibiotic and anti-inflammatory effects similar to the corticosteroids’ ones, but without side effects, as skin allergic reactions^15^^,^ 16. On cells, DPG is able to inhibit the hyaluronidase enzyme and, consequently, avoid the damage to the extracellular matrix, the histamine release and inflammatory chemical mediators, leukotrienes and prostaglandins^16^.

Experimental study using DPG on skeletal muscle pointed to its regenerative capacity, suggesting that DPG is able to modulate the satellite cells, induce myoblast and myotube differentiation and lead to muscle hyperplasia^17^. On glioblastoma cells, DPG controls tumor proliferation by inducing apoptosis through the inhibition of the nuclear factor kappa B (NF-kB), related to inflammation, mediated signaling pathway^18^.

Considering the cellular and molecular complexity mechanisms involved in the skin wound healing, the physiological and structural changes in the elderly skin that can contribute to the delay in repair, and the DPG properties, this research attempted to characterize its benefits in skin wound healing, through assessment the re-epithelization, changes in epidermis and dermis thickness, identification and quantification of dermic collagens I and III comparing adult and elderly skin over the 21 days of healing in animal model of excisional healing.

## Methods

### Dipotassium glycyrrhizinate cream

DPG (C_42_H_60_K_2_O_16_) was manipulated as a cream at the concentration of 2% (Table 1). It was supplied by Verdi Cosmetics (Joanopolis-SP, Brazil, 64.786.031/0001-00).

**Table 1:** Dipotassium glycyrrhizinate cream gel 2%.

Ingredient	%	Function	INCI Name	CAS Number
Aqua	93.600	Solvent	Aqua	7732-18-5
Sepigel 305	4.000	Viscosity controlling	Polyacrylamide, C13-14 Isoparaffin (and) Laureth 7	9003-05-8/246538-79-4/68439-50-9/9002-92-0/7732-18-5
Dipotassium glycyrrhizinate	2.000	Active	Dipotassium glycyrrhizinate	68797-35-3
Euxyl PE 9010	0.400	Preservative	Phenoxyethanol (and) Ethylhexylglycerin	122-99-6/70445-33-9

INCI: International Nomenclature of Cosmetics Ingredients; CAS: Chemical Abstracts Service.

### Experimental groups

The research was previously approved by the Ethical Committee for Animal Use (CEUA) of Universidade São Francisco (protocol no. 001.09.17) and Universidade de Taubaté (protocol no. 005/17), following the guidelines of the Brazilian Society of Laboratory Animal Sciences (SBCAL).

Wistar rats were supplied by Laboratory Animals Breeding and Commerce (ANILAB Laboratório de Diagnóstico Animal, Paulínia, SP, Brazil) and kept at the Animal Experimentation Vivarium of Universidade de Taubaté in individual cages, at 22 ± 3°C on a 12 h light/dark cycle, with free access to standard diet and *ad libitum* water.

Twenty male rats (Wistar) were used–10 adult animals (3 months old, weighing between 274-390 g) and 10 elderly animals (12 months old, weighing between 454-569 g).

### Surgical procedure

The animals were submitted to anesthetic procedure with 2% xylazine hydrochloride (Xilazin®, Syntec, Santana de Parnaíba, SP, Brazil) (10 mg.kg^-1^) associated with 5% dextrocetamine hydrochloride (Ketamin®, Cristália, Itapira, SP, Brazil) (25 mg.kg^-1^), prepared by combining 0.5 mL of xylazine (10 mg) with 0.5 mL of ketamine (25 mg) to a volume of 1 mL administered by intraperitoneally (1 mL.kg^-1^)^8^.

After trichotomy of the dorsal region and skin asepsis, each animal was submitted to two circular excisions, with 2 cm in diameter, 2 cm distant from each other, in median dorsal plane, limited in depth by muscle aponeurosis, performed with a scalpel (handle and blade number 15)^8^.

**Figure 1:**
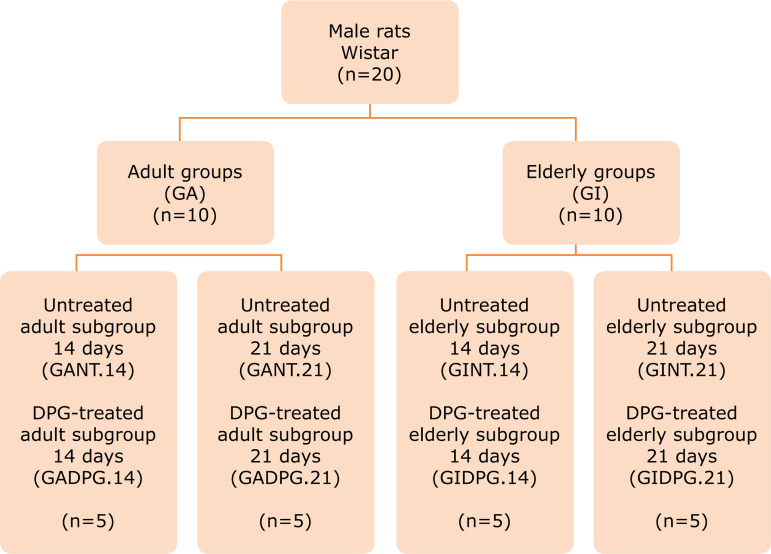
Fluxogram of experimental groups: adults (GA); elderly (GI); non-treated–GANT14, GANT21, GINT14, and GINT21; treated–GADPG14, GADPG21, GIDPG14, and GIDPG21.

The experiment was randomized into naïve (health skin), untreated (NT) or DPG-treated (DPG) animals and divided into groups following site (proximal or distal), treatment (untreated or DPG-treated) and day of euthanasia (14 or 21). After excision, the proximal wound was untreated, and distal wound was daily treated with 0.1 mL of 2% DPG cream, for seven days, starting 24 hours after surgery. Both proximal (untreated) and distal wound (DPG-treated) did not require bandages and healed by second intention, based on literature^8^, which used tretinoin for the same purpose. All animals were euthanized after the wound scar excisions at day 14 or 21, as described in Fig. 1.

- GAN (n = 10): the skin initially removed from adult (A) was used as naïve (N) group;
- GIN (n = 10): the skin initially removed from elderly (I) was used as naïve (N) group;
- GA14 (n = 5): skin (proximal and distal) from GAN (adult) group submitted to excisional scar *in vivo* at day 14. Those skin originated the groups GANT14 (untreated, proximal skin, euthanasia at day 14) and GADPG14 (DPG-treated, distal skin, euthanasia at day 14);
- GA21 (n = 5): skin (proximal and distal) from GAN (adult) group submitted to excisional scar *in vivo* at day 21. This skin originated the groups GANT21 (untreated, proximal skin, euthanasia at day 21) and GADPG21 (DPG-treated, distal skin at day 21);
- GI14 (n = 5): skin (proximal and distal) from GIN (elderly) group and submitted to excisional scar *in vivo* at day 14. Those skin originated the groups GINT14 NT (untreated, proximal skin, euthanasia at day 14) and GIDPG14 (DPG-treated, distal skin euthanasia at day 14);
- GI21 (n = 5): skin (proximal and distal) from GIN (elderly) group and submitted to excisional scar *in vivo* at day 21. This skin originated the groups GINT21 (untreated, proximal skin, euthanasia at day 21) and GIDPG21 (DPG-treated, distal skin euthanasia at day 21).

### Macroscopic analysis

The DPG-treated and untreated wound healing in both adult and elderly animals were evaluated throughout the experimental period, comparing the groups by macroscopy^8^. The initial area (large versus small diameter) was measured from day 0 to day 21 using a caliper (Universal Digimess 100003, Sao Paulo, SP, Brazil) and the scar tissue was removed on day 14^8^ (GA14: GANT14, GADPG14; GI14: GINT14, GIDPG14) or on day 21^8^ (GA21: GANT21, GADPG21; GI21: GINT21; GIDP21).

### Microscopic analysis

For microscopical analysis, the skin samples were fixed in 10% formaldehyde solution (Labsynth®, Diadema, SP, Brazil) for 24 h, attached at the end to cork, dehydrated in increasing ethanol concentrations (Labsynth®, Diadema, SP, Brazil), clarified in xylol (Labsynth®, Diadema, SP, Brazil), embedded in paraffin (Labsynth®, Diadema, SP, Brazil), and submitted to microtomy (Lupetec MRPO3, São Carlos, SP, Brazil).

The slides (sections 5-μm thick) were deparaffinized in two xylol baths (10 minutes each), hydrated in decreasing series of ethanol (100, 95, 80, and 70%) and distilled water, and stained with hematoxylin-eosin (HE) to measure epidermis thickness. Masson’s trichrome (MT) was used to measure dermis thickness, and picrosirius red (PR) to identify and quantify collagen fibers. After staining, slides were dehydrated in an increasing series of ethanol (70, 80, 95, and 100%) and xylol, and mounted with synthetic Canada balsam.

Epidermis, dermis thickness and collagen quantification were measured by computer-assisted image analysis method^8^^,^ 19 ^,^ 20 under an optical microscope. The image capture system consisted of a digital color camera Infinite-3C® (Lumenera, Ottawa, Canada) coupled to a microscope Nikon® a-photo-2-YSC (Nikon, Tokyo, Japan) connected to a computer (Dell®, Austin, United States; Pentium 4 processor, dual-core, 1.8 Mb, Windows XP® platform). Final magnification of 40x was used for epidermis and of 10x for dermis and collagen fibers. From each histological section, three different fields were analyzed. The images were sent and processed on a computer using the NIS for Windows program^8^.

### Statistical analysis

The analysis of the results was performed by adopting p < 0.05, to reject the null hypothesis using the following statistical tools: sample size, descriptive statistics, measures of central tendency, normality test, comparison test (*t* test) to homoscedastic distribution or Mann-Whitney to heteroscedastic distribution; ANOVA followed by Bonferroni post-hoc tests, partial correlation (Spearman’s test). The Statistical Package for the Social Sciences (SPSS) 20.0 (IBM Corporation, São Paulo, SP, Brazil) for Windows was used.

## Results

**Figure 2:**
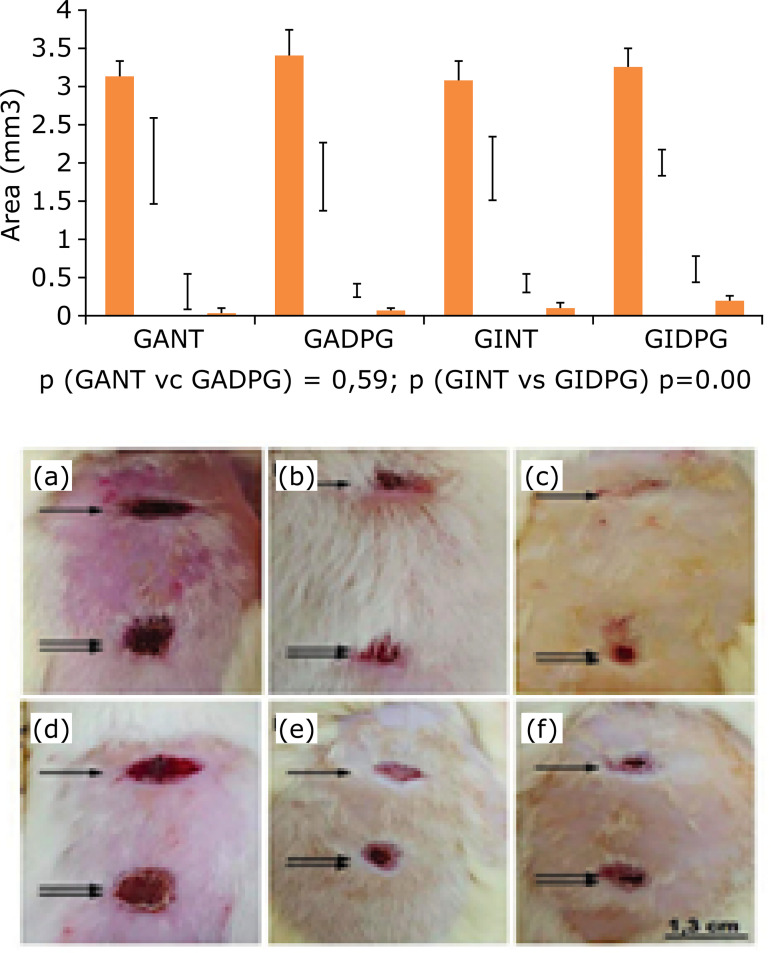
Graphical representation (groups GANT, GADPG, GINT and GIDPG; area in mm^3^) and macroscopical analysis **(a-f)** of wound healing overtime of skin resection [after 7 (a, d), 14 **(b, e)**, 21 **(c, f)** days]. Observe the macroscopic aspect in adults (n=10)**(a-c)** and elderly (n=10, Bonferroni post-hoc test) **(d-f)** animals, untreated (arrow) and treated (double-arrow) with DPG (bar=1.3 cm).

GANT: non-treated group adult naïve; GADPG: group adult treated with DPG; GINT: non-treated group elderly non-treated; GIDPG: group elderly treated with DPG; DPG: dipotassium glycyrrhizinate.

Histological analysis revealed intact skins for GAN and GIN. In both groups, epidermis presented a stratified squamous epithelium, with stratum basalis (basal cell layer), stratum spinosum (prickle cell layer), stratum granulosum (granular cell layer), stratum lucidum, stratum corneum (keratin layer), and dermis (papillary and reticular dermis) composed of connective tissue consisting of collagen containing blood vessels, hair shafts and glands, considering the specific aspects of an adult skin and an elderly one^5^, in which the arrangement between collagen fibers in the dermis is lost and the proliferation of keratinocytes in the stratum basalis decreases. After the experimental protocol, it is possible to observe the progressive closure of the excisional area, in adults and elderly animals, after 7, 14 and 21 days (Fig. 2).

**Figure 3:**
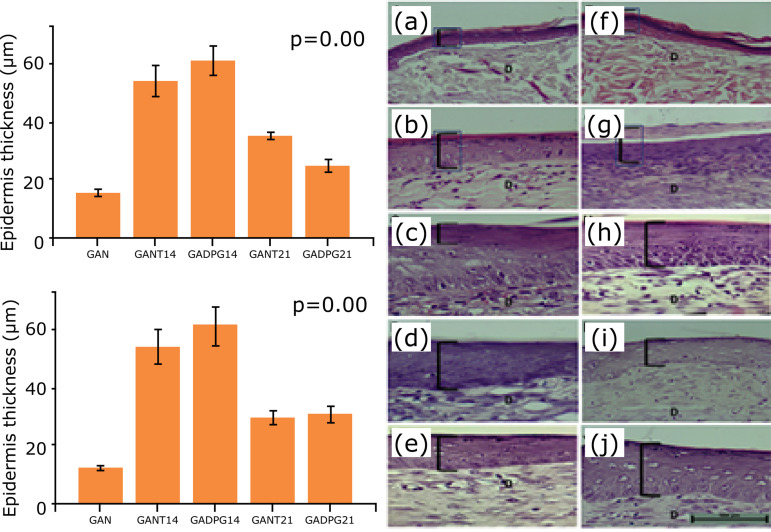
Graphical representation and histological analysis of epidermis thickness (groups GAN - **a**, GANT14 - b, GADPG14 - **c**, GANT21 - **d**, GADPG21 - **e**; GIN - **f**, GINT14 - **g**, GIDPG14 - **h**, GINT21 - **i** and GIDPG21 - **j**; bar=100μm). Observe the normal aspects in naïve **(a, f)** and regenerated **(b-e; g-j)** skins. Note the increase of epidermis thickness after DPG treatment in adult (n=10), Bonferroni post-hoc test (c) and elderly (n=10) (h, j) animals.

GAN: group adult naïve; GANT: non-treated group adult naïve; GADPG: group adult treated with DPG; GIN: group elderly; GINT: non-treated group elderly; GIDPG: group elderly treated with DPG; DPG: dipotassium glycyrrhizinate.

**Figure 4:**
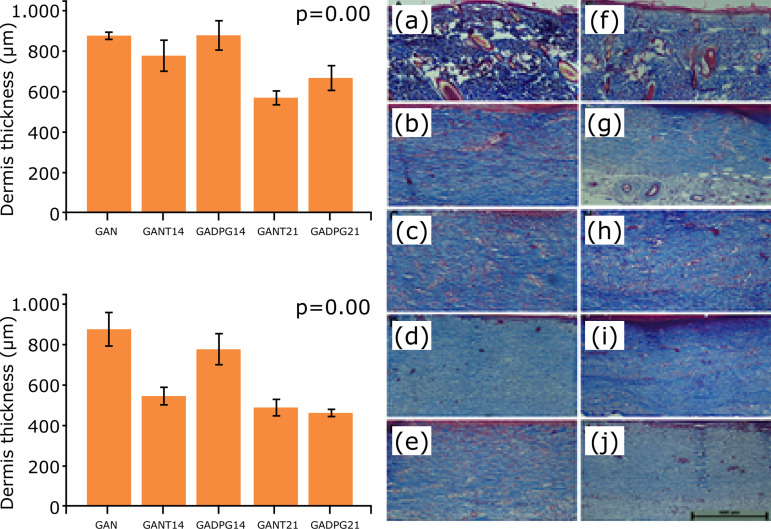
Graphical representation and histological analysis of dermis thickness (groups GAN - **a**, GANT14 - **b**, GADPG14 - **c**, GANT21 - **d**, GADPG21 - **e**; GIN - **f**, GINT14 - **g**,GIDPG14 - **h**, GINT21 - **i** and GIDPG21 - **j**; bar=100 μm). Observe the normal aspects and collagen distribution in naïve (**a, f**) and regenerated (**b-e; g-j**) skins. Cutaneous appendages were not observed in regenerated dermis (**b-e; g-j**). (n=10, Dunnett post-hoc test).

GAN: group adult naïve; GANT: non-treated group adult naïve; GADPG: group adult treated with DPG; GIN: group elderly; GINT: non-treated group elderly; GIDPG: group elderly treated with DPG; DPG: dipotassium glycyrrhizinate.

**Figure 5:**
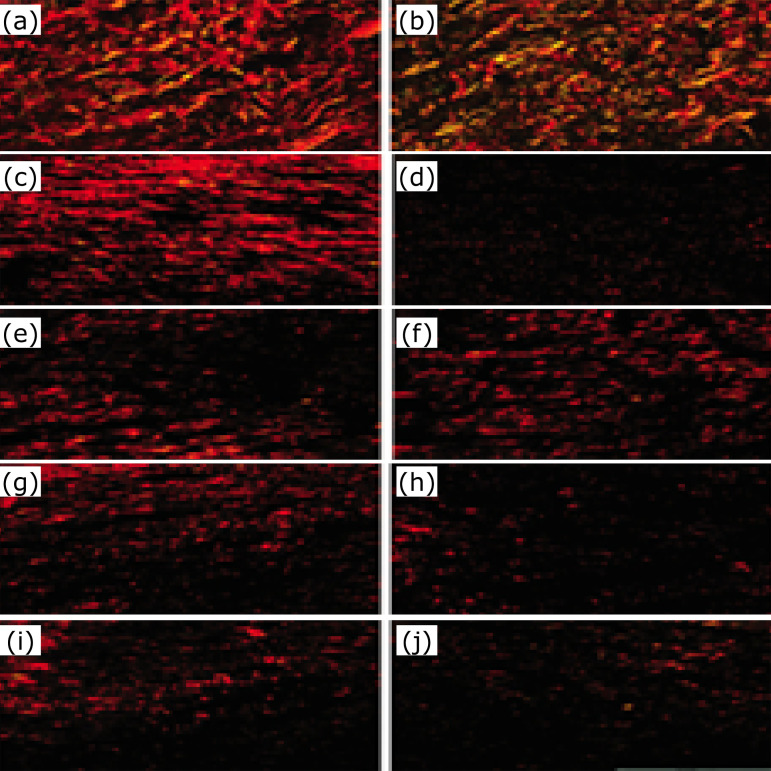
Graphical representation of collagens I (**a, c**) and III (**b, d**) distribution in the dermis of groups GAN, GANT14, GADPG14, GANT21, GADPG21, GIN, GINT14, GIDPG14, GINT21 and GIDPG21. Observe that the amount of collagen I is higher than the one of collagen III in adult (**a**) and elderly (**c**) animals. Note that the collagen III significantly reduced in (**b**) adult and elderly (**d**) animals after skin resection (n=10, Dunnett post-hoc test).

GAN: group adult naïve; GANT: non-treated group adult naïve; GADPG: group adult treated with DPG; GIN: group elderly; GINT: non-treated group elderly; GIDPG: group elderly treated with DPG; DPG: dipotassium glycyrrhizinate.

**Figure 6:**
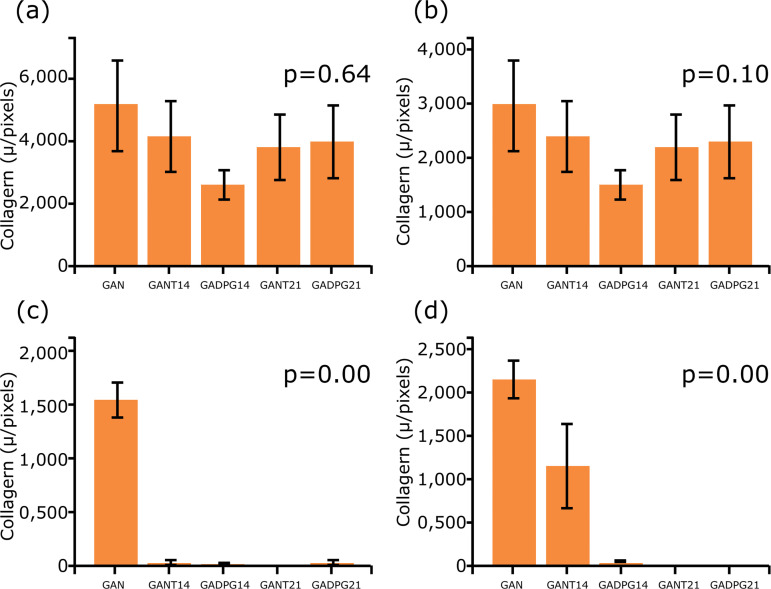
Histological analysis of collagen I (red) and collagen III (green) distribution in the dermis of groups GAN (**a**), GIN (**b**), GANT14 (**c**), GINT14 (**d**), GADPG14 (**e**), GIDPG14 (**f**), GANT21 (**g**) GINT21 (**h**), GADPG21 (**i**) and GIDPG21 (**j**) (bar=100 μm). (n=10, Dunnett post-hoc test).

GAN: group adult naïve; GANT: non-treated group adult naïve; GADPG: group adult treated with DPG; GIN: group elderly; GINT: non-treated group elderly; GIDPG: group elderly treated with DPG; DPG: dipotassium glycyrrhizinate.

The epidermis thickness of the regenerated skin, in adults and elderly animals, after 14 days, was similar between GANT14/GADPG14, and GINT14/GIDPG14. Regarding DPG effects in the regenerated skin, epidermis was thicker after DPG administration in adult and elderly rats when compared to naïve (GAN/GIN) skins (Fig. 3). For dermis, there were no significant changes in collagen I in adult animals, treated (GADPG) or untreated (GANT) (Fig. 4). However, higher collagen I density was observed in elderly animals, especially in those treated with DPG (GIDPG). Elderly animals, untreated or treated with DPG, decreased collagen III distribution (p= 0.027) (Figs. 5 and 6).

Regenerated skins in adults and elderly animals, after 21 days, displayed the same histological aspects of naïve skins in GANT, GINT, GADPG, and GIDPG groups (Figs. 3 and 4). For regenerated skins, the epidermis thickness was similar between GANT21 and GADPG21, and was thicker in GANT21, GADPG21, GINT21, GIDPG21 when compared to naïve skin (Fig. 3). For dermis, higher collagen I density was observed in GADPG21 when compared to naïve skins (Fig. 4). There were no significant changes in collagen I in adult animals treated (GADPG21) when compared to untreated ones (GANT21) (Figs. 5 and 6).

Comparing the regenerated skins after 14 and 21 days, the epidermis thickness was similar between GANT14/GADPG14, GINT14/GIDPG14, and GINT21/GIDPG21, being thicker after 14 days than after 21 days. Considering the epidermis of adult animals, there were significant changes in GAN *vs*. GANT14 (p=0.000), GAN *vs*. GADPG14 (p=0.000), GAN *vs*. GANT21 (p=0.006), GAN *vs*. GADPG21 (p=0.006), and GADPG14 *vs*. GADPG21 (p=0.000), although the epidermis was thicker in GANT21 than GADPG21 (p=0.02). For elderly animals, regarding epidermis, the difference was found when the groups GIN and GINT14 (p=0.001), GIN and GIDPG14 (p=0.001), GINT14 and GINT21 (p=0.01), and GIDPG14 and GIDPG21 (p=0.001) were compared (Fig. 3). Epidermis was thinner after 21 days of DPG administration.

Dermis was also restructured with papillary and reticular portions, but without skin appendages. It was thicker in GAN/GIN, when compared with treated or untreated dermis. After 14 days, dermis was thicker than after 21 days, especially in elderly animals (p=0.005) when compared to adults. Adults’ dermis thickness was similar among all groups. However, for elderly ones there were differences among GIN *vs*. GINT14 (p=0.001), GINT14 *vs*. GIDPG14 (p=0.045), GIDPG14 *vs*. GIDPG21 (p=0.003) groups (Fig. 4).

Under polarized light microscopy type I collagen, there were red-colored birefringence and type III collagen green birefringence. Type I collagen fibers were more abundant than type III collagen fibers in adult and elderly groups (Fig. 5).

There were no significant changes in collagen I in adult animals treated (GADPG) or untreated (GANT) at 14 and 21 days. However, adult animals showed differences on the collagen III when comparing the groups GAN *vs*. GANT14 (p=0.000), GAN *vs*. GADPG14 (p=0.000), GAN *vs*. GANT21 (p=0.000), and GAN *vs*. GADPG21 (p=0.000). In elderly animals, the amount of collagen I was similar between GIN and GIDPG14, but lower in GINT14. The opposite was observed after 21 days, when collagen I was higher in GINT21 than in GIDPG21. There was difference in collagen III among groups GIN *vs*. GIDPG14 (p=0.00), GIN *vs*. GINT21 (p=0.00), GINT14 *vs*. GIDPG14 (p=0.00), and GINT14 *vs*. GINT21 (p=0.026). After 14 days, elderly animals untreated or treated with DPG decreased collagen III distribution (p=0.027) (Fig. 6).

There are some associations between variables in the present study, such as: age is inversely related to collagen I (r=-0.474, p<0.01), and collagen III is inversely correlated to day of evolution (r=-0.467, p=0.01).

The mean and standard deviation found in each group are presented in Table 2.

**Table 2:** Epidermis and dermis thickness and collagens I and III quantification per experimental group * .

Skin	Day	Group	Mean	Standard deviation	p-value
Epidermis thickness (μm)	d0	GAN	15.24	2.52	0.000
		GIN	12.34	1.90	
	d14	GANT	54.13	12.40	
		GADPG	61.39	10.82	
		GINT	54.09	14.19	
		GIDPG	61.95	14.92	
	d21	GANT	35.60	2.92	
		GADPG	24.87	4.91	
		GINT	29.62	5.97	
		GIDPG	30.78	6.81	
Dermis thickness (μm)	d0	GAN	868.81	50.20	0.000
		GIN	1097.48	125.99	
	d14	GANT	598.02	138.72	
		GADPG	816.83	312.76	
		GINT	649.21	130.86	
		GIDPG	934.26	214.33	
	d21	GANT	566.46	74.19	
		GADPG	666.64	140.54	
		GINT	579.81	125.95	
		GIDPG	546.45	64.92	
Collagen I	d0	GAN	5.13	3.35	0.047
		GIN	2.65	1.56	
	d14	GANT	4.10	2.69	
		GADPG	2.52	1.01	
		GINT	1.19	0.89	
		GIDPG	2.65	2.32	
	d21	GANT	3.76	2.39	
		GADPG	3.89	2.93	
		GINT	1.35	0.80	
		GIDPG	0.60	0.30	
Collagen III	d0	GAN	1.53	0.41	0.000
		GIN	2.17	0.47	
	d14	GANT	0.026	0.056	
		GADPG	0.008	0.009	
		GINT	1.16	1.10	
		GIDPG	0.01	0.017	
	d21	GANT	0.0006	0.0005	
		GADPG	0.021	0.041	
		GINT	0.0001	0.0001	
		GIDPG	0.004	0.005	

* Data are reported as means ± standard deviation (analysis of variance); d0: day of excisional lesion; d14 and d21: euthanasia; GAN: group adult naïve; GIN: group elderly naïve; GANT: non-treated group adult; GADPG: group adult treated with DPG; GINT: non-treated group elderly; GIDPG: group elderly treated with DPG; DPG: dipotassium glycyrrhizinate.

## Discussion

The healing process is a physiological response to an injury and is marked by cell proliferation of the basal stratum of the epidermis and re-epithelization^1^^-^ 4 collagens I and III synthesis^4^^,^ 21 and dermal remodeling^2^^,^ 4 ^,^ 21.

In aging, important physiological and morphological events gradually occur, such as keratinocytes and fibroblasts apoptosis, collagen, elastin, proteoglycans and glycosaminoglycans synthesis decreation, dermal papillae shrinkage, thinner epidermis and dermis^5^^,^ 6, leading to a slow healing with functional and structural changes^6^^,^ 7.

In the last decades, research has been done to find alternative methods or new compounds that promote skin healing. Among them, retinoids, oils, and plant extracts have been studied. Such compounds were able to increase epidermis thickness^8^ and promote dermal tissue formation^8^^-^ 10.

Vegetable oils, such as *Copaifera langsdorffii* 22 and *Caryocar coriaceum* Wittm^23^, *Passiflora edulis* extract (passion fruit)^24^ and *Carissa spinarum* Linn methanolic extract^25^, as well as DPG, were able to significantly accelerate the reduction of the injured area, increasing the rate of wound contraction and re-epithelization.

Such processes were observed after induced injury in the experimental groups studied. Specifically, when considering re-epithelialization of the affected area and epidermis thickness, it was effectively better in the groups treated with DPG. Differences in epidermal thickness are greater at 14 days than at 21 days in both adult and elderly treated animals. At 21 days, reduction in the thickness of the epidermis of the animals was observed at similar levels to the naïve animals.

The mechanism of re-epithelization by DPG is similar to the one of other compounds, such as leptin^26^, sericin^27^ and curcumin^28^, with significant proliferation, differentiation and migration of keratinocytes, increasing the thickness of the epidermis. Similar effect of DPG was observed in muscle regeneration studies, in which DPG induced differentiation of satellite cells in myoblasts and from these in myotubes, with formation of fully regenerated fibers early, after seven days^17^. These results suggest that DPG has a positive modulating effect on cellular hyperplasia, probably by activation of the NF-κB pathway, in a mechanism opposite to that one described for glioblastoma cells^18^.

Regarding the dermis, DPG induced the formation of a totally restructured extracellular matrix dermis, without signs of fibrosis. This is probably due to its inhibitory effect on the hyaluronidase enzyme and, consequently, on hyaluronic acid^16^.

Studies have shown that lipoic acid (LA)^29^, 18β-glycyrrhetinic acid (18β-GA)^30^ and tretinoin act significantly in the repair of the dermis, increasing the number of fibroblasts and the collagen synthesis^8^. Such results were also observed after the use of collagenase and trans-retinoic acid^9^^,^ 10, as well as DPG.

Collagen is the main structural protein produced by fibroblasts and makes up the extracellular matrix in normal tissue, becoming the most abundant protein during skin healing^31^. Normal dermis contains approximately 80-85% of collagen I and 10-15% of collagen III^32^^,^ 33. Collagen I are thick-stable fibers that guarantee the resistance of the tissue to mechanical forces, while collagen III are fine-fibrils, arranged below the basement membrane, that guarantee the adherence of the epidermis to the dermis^34^.

Skin is affected by photoaging, decreasing the levels of collagens I and II^32^^,^ 36, mucopolysaccharides and elastin and increasing the activity of collagenase^35^^,^ 36. In the skin healing process, collagen III synthesized during the proliferative phase is commonly replaced by collagen I during tissue remodeling phase^37^.

In this study, the presence of collagen I was observed in adult and elderly animals, after 14 and 21 days, treated or not with DPG. The amount of collagen I was higher in adult animals than in elderly animals, as expected. However, after 14 days, the effect of DPG on the dermis of elderly animals led to an increase in collagen I density to levels similar to the ones of naïve animals.

Increases in collagen III density levels were found in the deep dermis in hypertrophic scars^38^ and keloid scars^39^, diverging from the present findings, in which type III collagen density was reduced even in naïve animals and was even lower in all other experimental groups. The associations between age and collagen I and collagen III and day of evolution suggest decreasing of collagen type I occurs because of the age, and there is reduction of collagen II in wound healing process.

Comparing the evolution of healing between adult and elderly animals over the healing period (0 to 21 days), and considering the pharmacological properties of DPG, the compound acted during healing, accelerating the regenerative process, especially in elderly animals. The results show a possible therapeutic potential of DPG, for the pharmaceutical and cosmetic industry, in wound healing by second intention, leading to re-epithelization and formation of a new dermis, rich in type I collagen.

## Conclusions

DPG promoted more effective epidermis and dermis proliferation and hypertrophy in the first 14 days, including the period of use (first seven days), in adult and elderly animals. Its continuous application could improve healing. Regarding collagen I synthesis, it was higher in adults, after 21 days, and in elderly, after 14 days, in detriment to the synthesis of collagen type III, without inducing fibrosis. The results suggest that DPG rebuilds dermis in a way similar to undamaged conditions.
